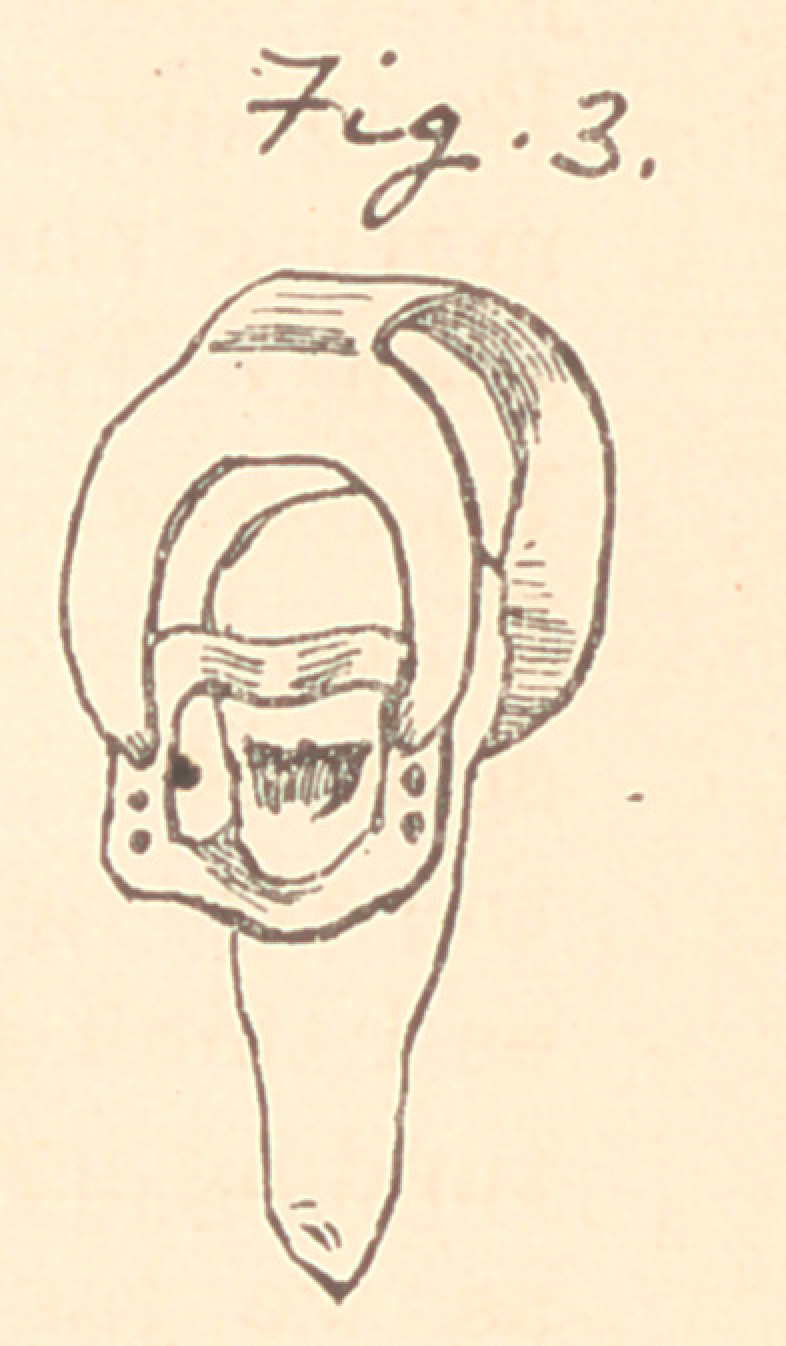# New York Odontological Society

**Published:** 1890-07

**Authors:** 

**Affiliations:** New York Odontological Society


### NEW YORK ODONTOLOGICAL SOCIETY.

THE New York Odontological Society held its regular monthly meeting Tuesday evening, March 18, 1890, in the New York Academy of Medicine, No. 12 West Thirty-first Street.

The President, Dr. J. Morgan Howe, in the chair. The Corresponding Secretary read the following communications:

"CHAS. F. IVES, M.D.S., *Corresponding Secretary, etc.*:

"MY DEAR SIR,—Your favor of the 20th, enclosing a copy of a preamble and resolution passed by the New York Odontological Society, at a regular meeting of said society, held on the 18th inst., is at hand.

"In answer, we wish to state that the author of said preamble and resolution has, either through ignorance of histological nomenclature or the English language, made a sad mistake, as neither of us, in any of our writings, has 'claimed the demonstration of the presence of a protoplasmic reticulum in both enamel and dentine,' or in either one of them.

"We would like further to state, that what is meant by 'three experts' in this branch of histology we are unable to understand, as we have no knowledge of any such.

"Very truly yours,

"C. HEITZMANN,
"C. F. W. BÖDECKER,
"FRANK ABBOTT."

"60 EAST FIFTY-EIGHTH STREET,
March 1, 1890.

"DR. C. F. IVES:

"DEAR DOCTOR,—Yours of February 28 at hand. In regard to Dr. Allan's resolution, I will submit to everything Dr. C. Heitzmann may agree to. Although I do not see that this matter can lead to anything else but a great deal of unpleasantness and harm for the Odontological Society.

"I am of the opinion that the matter is a purely personal one between Drs. Heitzmann and Allan, and should be settled by them.

"Yours truly,

"C. F. W. BÖDECKER."

On motion of Dr. Jarvie, the communications read were received and placed on file.

*The President.*—The next order of business will be Incidents of Practice and Casual Communications. Under this head, I will present and describe a device for retaining the dam in place while operating on cervical cavities. It consists of a face piece, so shaped as to hold the dam above the cavity, and to be itself held in position against the labial or buccal surface of the tooth by a clamp or other means. I have used this device for about two years, postponing the public presentation of it because I realized that it was imperfect. I have recently made an improvement in the form of the face piece, and present it now, in the hope that some one will develop the idea more perfectly. The way in which I have worked this idea out thus far is to make the dam-retainer or face piece of thin steel. (I have used French, cold-rolled steel, twenty-nine or thirty gauge.) It will pass around two teeth with the dam-retainers held in place upon them by clamps. The shape and size of a face piece or dam-retainer, suitable in most cases for use on the ten anterior teeth, is shown in Fig. 1. A series of small holes are drilled at either side. At one edge of the central aperture a concavity is filed to fit the neck of the tooth, and this edge is turned over to nearly a right angle with the plane of the other parts (as at *a*). On the opposite side of the aperture (*b*) the surface is made slightly concave (on the flat) where it rests on the coronal surface of the tooth. The form can be variously modified to suit special cases, but this is the shape found most useful for general application. The most convenient device for holding this retainer on the tooth which I have yet contrived is this clamp, having two points to engage in either pair of holes in the retainer, and one square end at the other extremity to bear on the neck of the tooth on the lingual surface. Fig. 2 shows this clamp. The opposing bearings (at *a a* and at *b*) exert pressure at exactly opposite points on the tooth, so there is no tendency to slip, while the retainer is held in its position, either high or low, equally well. The dam-retainer, held on a bicuspid by this clamp, is shown in Fig. 3. In some difficult cases, a very small piece of modelling compound may be placed on the part of the face piece which is to rest on the crown of the tooth (Fig. 1, *b*), and while the compound is soft, place the retainer on the tooth in the position in which it is desired to be held, holding it in place with the finger while the clamp is applied. When a tooth is somewhat separated from its neighbors, the face piece can be tied fast with silk or thread. The dam is to be applied after the retainer is adjusted, and I have found thin rubber to answer best. So far as I know, the idea is free and unpatented, and I describe, show, and illustrate it in order that it may be free not only to use as it is now, but free to improve on without let or hinderance.

We will be very happy to hear from any one present who has anything else to offer under the head of Incidents of Practice or Casual Communications.

*Dr. Edward S. Niles.*—I have recently been interested in an apparatus for destroying or obtunding the sensitiveness of dentine; and just as I was about leaving home the man who patented this apparatus was in my office, and I said, If you will loan me one of your appliances to take to New York I will give it to some of my professional friends there to test. He consented, so I brought it with me. I left it with Dr. Bogue, who suggested that it might be of interest to this meeting. I will give you first the history of the apparatus. It is an invention of a man in Providence, R. I. First used for this purpose about a year ago, it is an outgrowth of an apparatus used in the practice of medicine to keep certain parts of the body at a given temperature, either by moist or dry heat. That is an entirely different appliance from this, and especially designed for use in the practice of medicine. The principle of this apparatus is simply the application of steam to sensitive dentine, and is very simple. By means of an alcohol lamp under this small boiler, steam is generated and forced through this small tube, drawn to a point about equal in diameter to that of a hypodermic syringe. This fine jet of steam is applied to the cavity for about five to fifteen seconds. By longer application it has been used for destroying pulps and removing pulps of teeth. My personal experience with it has been slight. There are members of the profession in Providence and Boston who have used it for several months with very good results. I am, however, so well pleased with it that I have rented an instrument. It accomplishes the end with very little pain if one has the experience to use it properly. I simply present it, gentlemen, as I think it is one of the coming things that we may need for obtunding sensitive dentine. It opens up a large field for experiment, as we can use warm applications of various solutions, alcohol, cocaine, or a large variety of things, which, in cold solution, would have little or no effect, may be made valuable as obtundents if vaporized and applied hot or warm.

*Dr. Lord.*—What is generally used?

*Dr. Niles.*—Water is generally used in the boiler.

*Dr. S. G. Perry.*—I am amply repaid for coming here to-night by one sight of this little clamp, which, I understand, is a device of the President. I have never seen anything of that kind before that I took very much interest in. It is a little risky for me to praise anything that I have not tried, but this seems to be a very ingenious little device, and so mechanically correct that it must succeed. I have never seen one that could be easily adjusted and that would stay in place; but made on this principle it is bound to stay every time.

*The President.*—Some teeth are so irregular that it will not keep its place well.

*Dr. William Jarvie.*—Obtunding the sensitiveness of dentine can, I think, be accomplished in a very much simpler manner than with the device presented to-night. Let me relate a case in which I employed it to-day. The cavity was situated on the labial aspect of the second inferior bicuspid, and was so sensitive that the touch of an instrument would make the patient shrink and suffer a great deal of pain. I applied the rubber dam, and with the hot-air syringe dried the cavity perfectly, then with a sharp instrument I excavated the cavity with almost no pain. I think the perfect dryness of the cavity and a sharp excavator are the best and safest obtunders we can have.

*Dr. Dwinelle.*—The instrument that has been presented to-night reminds me of a similar one introduced to the profession years ago. It had a pendulous lamp underneath, like this, for heating the air to the right degree. It was rather a bungling affair, and very much like many other complex instruments which are made to accomplish a simple purpose. It reminds me of an anecdote of one of the Khedives of Egypt, who had a pair of gold snuffers presented to him. He hardly comprehended how to use them, but in a measure the idea finally dawned upon him, and taking the snuffers, he opened them, snuffed the candle with his thumb and fingers, then put the results into the snuffers, threw up his hands, and said, "Great is Alla, and Mahomet is his prophet!" A great many intricate and useless things accumulate in our laboratories. The hot-air pendulous lamp referred to I have in my museum still, which is filled with all sorts of devices for doing things that we can often do at our fingers' ends in a moment. With these devices there is often but little relation between cause and effect, it is like shooting a mosquito with a ten-pound cannon! In reference to obtunding hypersensitive dentine I have very little difficulty in the matter. Hot air is quite sufficient in most instances, but in ninety-nine cases out of one hundred I use simple chloride of zinc. I recommended it to the profession a number of years ago. Sometimes it produces a little pain for a few minutes, but usually nothing to speak of. I apply it with perfect audacity and impunity under all circumstances. I never devitalized a pulp with it in my life. Sometimes I have used it in a somewhat heroic sort of way. I have dried my cavity and filled it full of the salt of chloride of zinc, and then, in order to enforce and project it into the sensitive dentine, I have applied a heated instrument to it; and without any subsequent trouble I think the sensitiveness of dentine can be very easily overcome with this agent.

*Dr. Cook.*—How does the patient feel about it?

*Dr. Dwinelle.*—That is a very important question to ask. Perhaps in the majority of cases there is some pain, but oftentimes little or none; as the patients say, "none to speak of." The philosophy of it is simply this: We actually destroy for the time being the sensitive fibrillæ projecting from the nerve itself, the fibrillæ in the dentinal tubuli. We have a great many cases of hypersensitive dentine which are very serious, especially at the cervical points of the teeth. I have had people come to me who could not breathe sidewise into the mouth upon the teeth without pain. One of my patients told me that in drawing in her breath the shock was so great that she positively dropped to the floor and was supposed to be in a fit. There was no erosion apparent in this case. In applying the chloride of zinc to these sensitive places by the gum, I first put on the rubber dam, adjust it to its place so as to draw the gum up to the periosteum, then apply the pure salt, and enforce it with a heated instrument. Sometimes, in a couple of weeks the patient has come back for a renewal, but very rarely after the second application. I have great confidence in the efficacy and safety of chloride of zinc. I am perhaps like the shoemaker who thought there was nothing like leather!

*The President.*—Gentlemen, I have the pleasure of announcing that Dr. G. L. Curtis, of Syracuse, is with us to-night, and will read a paper, the title of which is "Local Anæsthesia by Nitrous Oxide."

(For paper, see page 403.)

*The President.*—Gentlemen, the subject of Dr. Curtis's very interesting paper is before you. I hope the gentlemen who are interested in this subject of sensitive dentine and its anæsthesia will take part in the discussion as promptly as possible.

*Dr. Curtis.*—A patient has been provided, I believe, whereby the use of this agent can be demonstrated before you this evening; and after the discussion, I will demonstrate the value of nitrous oxide as an obtundent of sensitive dentine.

*Dr. Bogue.*—Do I understand Dr. Curtis to announce as his belief that dehydration, or getting rid of the water in the dentinal tissue, is the most effective method of getting rid of the sensitiveness?

*Dr. Curtis.*—I believe it is the most complete method.

*Dr. Bogue.*—Further, I would ask whether his experiments show any special difference in the results obtained by whatever process the tissues are dried or the water got rid off; because we have a number of agents that have a very strong affinity for water, and we have hot air as well as nitrous oxide.

*Dr. Curtis.*—I do not think it would make any difference in what way the water is disposed of, as to the sensitiveness; but it does matter what agents are used as to the effect upon the tissues. I mean that some of the agents employed have a very injurious effect upon the tissues, and their use is not warranted.

*Dr. Bogue.*—Would you kindly mention them.

*Dr. Curtis.*—Carbolic acid, chloride of zinc, hot air, alcohol, sulphuric acid, etc.

*Dr. Bogue.*—How about glycerin?

*Dr. Curtis.*—I have never used it. It will dehydrate.

*Dr. Bogue.*—How about absolute alcohol?

*Dr. Curtis.*—It will also dehydrate. I believe alcohol will produce subsequent inflammation. Its use is followed by pain.

*Dr. Perry.*—I have a cylinder in my cellar in which I condense air. We keep it on tap, at from twenty-five to fifty pounds pressure. I would like to ask Dr. Curtis whether we might not expect to get the same result from that condensed air that he gets with nitrous oxide?

*Dr. Curtis.*—I do not think a pressure of fifty pounds sufficient to produce the desired effect. High pressure is essential. The rapid expansion of nitrous oxide produces increased coldness over that of air. Where the dehydration occurs, the moisture is taken up and carried off.

*Dr. Perry.*—Would you get the same effect by using air alone under the same pressure? You claim that there is nothing especial in the gas itself.

*Dr. Curtis.*—I think not, as air is not as expansive, consequently could not be as cold.

*Dr. Dwinelle.*—You do not claim that the nitrous oxide has any sedative effect?

*Dr. Curtis.*—No, sir.

*Dr. Dwinelle.*—You get the coldness from the great degree of evaporation produced.

*Dr. Curtis.*—Rapid evaporation.

*Dr. Bogue.*—I am glad that Dr. Curtis's experience is what it is, for, in continuing some experiments begun some six or eight years ago with veratria dissolved in absolute alcohol, to which an equal volume of glycerin was added, I happened to stumble across the idea of adding an equal volume of cocaine, carbolic acid, and tannin, and I found almost the same results that Dr. Curtis speaks of. I mention it in case some of my professional brethren could not easily get a cylinder of nitrous oxide. This you can get.

*Dr. Perry.*—Is that successful?

*Dr. Bogue.*—It is in the majority of cases.

*Dr. Perry.*—What is the formula?

*Dr. Bogue.*—Veratria, such quantity as you please, dissolved in absolute alcohol, to which add an equal volume of glycerin and carbolic acid. A few months since, I took cocaine, *quantum sufficit*, dissolved it in absolute alcohol, and added tannin to saturation; to this I added carbolic acid and glycerin, an equal volume of each; the same idea being present that Dr. Curtis has advocated. Of these two mixtures I take equal quantities, mix them together, and put them into the cavity. If I put it into a large cavity and go to work at a small one, by the time I have finished the small one the other is pretty near devoid of its sensitiveness.

*Dr. Perry.*—What element of danger is there in the remedy?

*Dr. Bogue.*—Veratria.

*Dr. Perry.*—Is it to be very carefully guarded against?

*Dr. Bogue.*—Of course, one-fifty-second of a grain is a dose.

*Dr. Ottolengui.*—I have paid a great deal of attention to the obtunding of sensitive dentine, and I want to say something about this theory of dehydration, because that is the theory on which I have worked. In the very beginning of the evening, before the paper was read, one gentleman announced that the obtunding of sensitive dentine was easy; that it is only necessary to dry out the cavity. That is it exactly. Dry it out. If it can be dried out with a hot-air syringe, that is all that is necessary. But it does not always follow that the simple application of heat from a rubber bulb will be sufficient to dehydrate the cavity. Consequently it may be advisable to do as Dr. Dwinelle did, use chloride of zinc crystals, because that absorbs more moisture. Chloride of zinc, in a fluid form, has attracted to itself moisture from the atmosphere, and therefore cannot take as much moisture from the tooth-tissues as the crystals. I got as far as that. I found a patient, who lives on Staten Island, who could not have a tooth filled, or even the old oxyphosphate taken out, the hot air not being sufficient to relieve the sensitiveness. Somebody, somewhere, whispered dehydration, and I at once saw that that is the result obtained whether we use hot air, chloride, or zinc. Then I conceived the scheme of intensifying the dehydration, and began to use an ether spray. I kept a record of cases for nearly two years, tabulating two hundred and fifty of them, and obtained success in every case, but attended by objectionable features in many cases, showing that dehydration was all right and the ether spray sometimes wrong.

I might say here, incidentally, that I thought at that time that I was originating an idea, but somebody told me I had not, for he had seen it in the *Cosmos*. I looked over the *Cosmos*, and found that about twenty-two or twenty-three years ago a very modest gentleman had suggested that it could be done in that way. I rarely use ether now except in extreme cases; but it does produce anæsthesia,—absolute anæsthesia; not as the hot-air blast does, nor as does the chloride,—*sometimes*,—but every time. When I remembered that Dr. Curtis told me, two or three years ago, that he had discovered the whole theory, I made up my mind that this was the place to come to-night. If what he says is true, nitrous oxide is our friend. Dr. Rhein uses chloride of methyl. I do not think there is any particular property in chloride of methyl except the extreme cold, but the cold is intense and the obtunding is very rapid. The question has been asked to-night whether it is the nitrous oxide gas only that is effective, or whether it is the high pressure. On the other hand, I could not help thinking, when the doctor described that case, that he had a little more than local anæsthesia; that relaxation of the muscles was caused by inhalation of the nitrous oxide. I have seen a gentleman produce anæsthesia in a tooth by simply waving a napkin saturated with chloroform over the mouth. In the earliest stages of anæsthesia, sensitiveness departs from the extremities.

I made, some two or three years ago, a long series of experiments with nitrous oxide gas, myself being the patient in order to note the stages of the anæsthesia. I made the experiments in this way: I would have an assistant prick me with a needle while the drug was administered, and I found invariably that I was conscious of the fact that sensation had ceased; proving that the first effect of anæsthesia is that sensation is controlled. It may be a little better to use nitrous oxide than hot air, because if any of the gas goes down the throat, so much the better; a little of it is very good; so if we are seeking for a powerful agent for dehydration, I have no doubt that a most convenient agent is nitrous oxide, as suggested by Dr. Curtis.

*Dr. Niles.*—As a matter of fact, a dehydrated nerve does not communicate sensation. This can be easily demonstrated by experiments on the frog. Those of us who have worked in the physiological laboratory know that frogs are used to demonstrate the reflex action of the spinal nerve. The medulla is severed, the nerve is dissected out of the frog's leg, and if the foot is irritated, the leg is quickly drawn up. If the nerve become dry, there is no movement on the same irritation, but remoisten with salt water, and the function returns. The only difficulty is in getting a cavity dry quickly enough so that the patient shall not suffer pain during the operation. It is very singular that I should be here with this apparatus, which produces insensibility in a manner exactly opposite by moist heat. It leaves one to suppose that heat alone is capable of producing insensibility of tooth-structure.

*Dr. Ottolengui.*—May I suggest that it operates on the same plan? Both methods produce heat, which seems to be the effective agent. The tooth, which is being operated upon, is so hard that no moisture is likely to get into the tooth-substance, and the heat that goes with the moisture will dehydrate the portion below the surface, although the surface itself is wet.

*A Voice.*—Is it not cooked?

*Dr. Niles.*—No, it is not cooked, because sensation returns to that point, which could not happen if it were cooked.

*Dr. Jarvie.*—It seems to me that there is a question behind all this that we should take into consideration. Many means may be used that will obtund the sensitiveness of teeth, but is it well to employ them, in view of the after-results? I think that when Dr. Curtis read the description of his throwing a blast of nitrous oxide into a cavity he gave us the best illustration why it should not have been done. He told us that the sensitiveness was completely obtunded; that he exposed the pulp which bled, and that he went on with the operation, capped the pulp and filled the cavity. There is great danger of obtunding teeth to a too great extent. I think it is wise to let the pulp have its normal degree of sensibility left as a guide to tell us not to cut too near it. Painless work is all very nice while excavating, but that is of little real advantage if, as a result, the filling be made too near the pulp, and inflammation and suppuration ensue.

I do believe the plan suggested, of drying out the cavity with a hot-air syringe, will obtund the sensitiveness sufficiently to enable the patient to bear the cutting, if sharp excavators are used. It is better for the patient to bear a little pain than to run the risk of exposing the pulp by wholly destroying, temporarily, sensation in the tooth. My experience is that a blast of cold air, such as the essayist recommends in a sensitive cavity, causes more pain than will be compensated for by the subsequent dryness.

*Dr. Mills.*—I want to speak of some experiments that I have been making in regard to obtunding sensitive dentine. I do not propose to say very much about what I have been doing, except to give you a statement of the facts. We all know that with all the suggestions and formulas that we have had in regard to the treatment of sensitive dentine, the howl goes on all over the country about the pain we cause. There is no profession that has so much ill-repute attached to it in regard to the matter of pain as ours. We are considered harsh and cruel by the people we try to serve, and it seems to me that, with all our remedies, there is still something to be done to correct this defect, or we are certainly in an unfortunate position. What I have been doing in this line I think you will admit is something new, when I tell you what it is. The result of my experiments has been very gratifying to both myself and my patients, and I have received many compliments in reference to it as compared with the treatment of others. I make use every day in my practice of different potential acids. I use sulphuric acid, nitric acid, and muriatic acid, for controlling sensitive dentine; and I am ready to demonstrate and prove their efficacy to any gentleman who may come to see me in my office at any time.

*Dr. Dwinelle.*—While I was on the floor, a moment ago, the query was made whether there was any considerable pain connected with the application of chloride of zinc. I admit that there is at times, but not as much or often as one would suppose. By applying the salt, pure and simple, and allowing it to deliquesce (by absorption), it is far less painful than when used in a liquid solution. In extreme cases I have qualified it with great success by drying the cavity and introducing cocaine for a short time and then applying the caustic. In such cases the patient never complains of pain. I sometimes use alcohol and other remedies, but chloride of zinc is my sheet-anchor, and I seldom have occasion for any other agent for obtunding sensitive dentine.

Perhaps I may have thrown a little ridicule upon the instrument that has been presented to-night, and I wish to say that I did not refer to that instrument especially. I do not say that the use of this apparatus is not effective, but only that simple measures will generally accomplish the end that is supposed to be accomplished by this somewhat complicated machine. I am greatly obliged to Dr. Niles for bringing this instrument to our attention, and I believe it will do good. I do not propose to ridicule anything that is the result of honest effort to advance our profession in the right direction.

With reference to the remark of Dr. Ottolengui, that he supposed he had invented or discovered a certain method of practice that was highly efficacious, and subsequently found that it had been used many years ago. I do not think that that militates against the actual discovery by him. I believe in coincident discovery. It is perfectly well known that important discoveries have been made simultaneously throughout the world by different individuals at almost the same hour. New planets have been discovered by different astronomers in different parts of this globe on the same night. It seems to be a principle in nature that when the world is in the most need of a great discovery, when she is virtually in travail for it, so to speak, that out of the laboratory of heaven, or the great mind of the Creator, the idea is vouchsafed to us, is sent down to us, and is received in various lands and by different minds at about the same time. So I am inclined to think that the fact that a man's discovery has been used before him is no evidence that it was not an original discovery on his part. It is possible that in the annals of eternity it may be proven that every thought is but a reproduction of some thought that has been produced and reproduced over and over in the history of the world of intelligence. So the matter of plagiarism is not so very remarkable after all. It is with caution that we should undertake to say that this man or that man has been guilty of plagiarism. History constantly repeats itself, and ideas are constantly repeated, so it is well for us to use charity in this matter. Good old Solomon said, thousands of years ago, that there was nothing new under the sun!

Dr. Curtis's paper is an exceedingly interesting one. It is not so very important to me in my own practice, but it may be hereafter. It has opened a field of inquiry in a direction that will prove profitable, and I certainly thank Dr. Curtis for his presentation of this subject to us. It will elicit a great deal of earnest and honest inquiry, and I have no doubt will lead to the development of that which we are aiming after.

*Dr. Brockway.*—This matter of obtunding sensitive dentine is not such an important one to us now, it seems to me, as it was a few years ago. I am led to make this remark from the fact that in my own practice I have far less complaint from my patients in regard to painful operations than I used to have. I do not doubt the efficacy of the methods described and the agents that have been used; and I am struck more forcibly than ever with the fact that "there is nothing new under the sun," when I find that what I supposed I discovered some years ago, a method of obtunding sensitive dentine by dehydration, had been discovered by some one and published a few years before. And it has been discovered by Dr. Ottolengui now, and doubtless will be discovered by many others in the future. I wrote, and read before the Brooklyn Dental Society some twelve or fifteen years ago, a paper on this subject of obtunding sensitive dentine by dehydration. I was led to the discovery in this way: From using the rubber dam to cover several teeth in which cavities were to be prepared, I found that the cavities last prepared were usually quite insensible to pain. Those cavities were in teeth that had been kept dry for some time by the application of the dam; and taking the hint from that, I improved upon it by using a jet of hot air from a syringe. I used this method quite extensively for some time, and occasionally use it now, but not as much as formerly,—for two reasons: one is that I was led to suspect that it might possibly be injurious to the tooth to keep it dry so long, or to dry it so thoroughly as I did. The other reason is that I have changed my method of preparing cavities largely. Instead of cutting them out dry, or in a half-dried condition, as formerly, I almost invariably nowadays have a jet of water thrown upon the bur while I am using it. I prepare cavities very largely with the engine, driven by a water motor, and using very sharp burs. No bur should be used unless it is *sharp*. With a jet of water thrown upon the bur, to keep it from heating and to wash away the chips, I can prepare cavities much more rapidly than formerly, and without causing excessive pain. I have the impression that much of the pain, where the bur is used, comes from the incautious use of it, from the heat produced by friction.

I do not know that I have anything more to say except to set myself right in the matter of the dehydration of cavities. I was not aware when I wrote the paper I speak of that the subject had been mentioned before.

Dr. Curtis here demonstrated successfully the use and advantage of his nitrous oxide blast by applying it to the teeth of a young man. The cavities were situated in the approximating surfaces of the central and lateral incisors. The teeth were exceedingly sensitive, and gave unendurable pain when an attempt to excavate the cavities was made before the gas was used. The rubber dam was adjusted and the blast gently applied for about two minutes, when the cavities were quickly and freely excavated without the slightest expression of pain or discomfort to the patient. Five minutes later the teeth were as sensitive as before the application of the nitrous oxide. The cavities were filled with gutta-percha.

*Dr. Ottolengui.*—I know that a number of gentlemen throughout the country are using my method of the ether spray, and I would like to say, after seeing Dr. Curtis's method of dehydration here, that I believe they will get better results with it than they do with the ether spray, though I believe that chloride of methyl is even better, if it can be obtained.

*Dr. Dwinelle.*—Mr. President, I presume I express the sentiments of every one present when I say that Dr. Curtis has certainly demonstrated all that he has claimed in reference to the new method of local anæsthesia; and I would move that a vote of thanks be extended to Dr. Curtis, not only for his very interesting paper, but for his successful demonstration.

Dr. Dwinelle's motion was carried.

Adjourned.

S. E. Davenport, D.D.S., M.D.S.,
*Editor New York Odontological Society.*

## Figures and Tables

**Fig. 1. f1:**
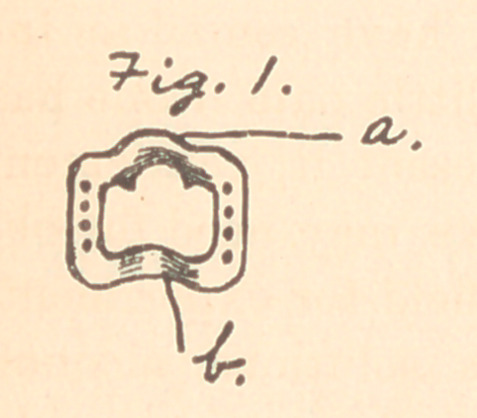


**Fig. 2. f2:**
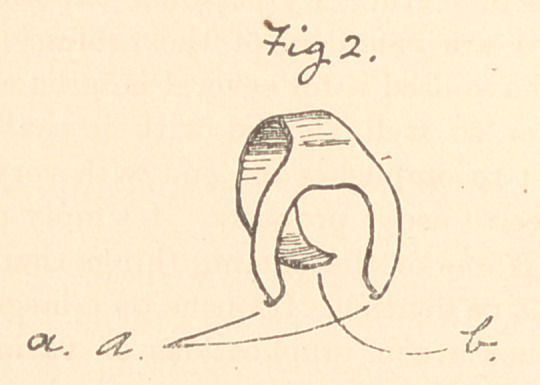


**Fig. 3. f3:**